# Motion artefact removals for wearable ECG using stationary wavelet transform

**DOI:** 10.1049/htl.2016.0100

**Published:** 2017-06-14

**Authors:** Shuto Nagai, Daisuke Anzai, Jianqing Wang

**Affiliations:** Nagoya Institute of Technology, Nagoya 466-8555, Japan

**Keywords:** electrocardiography, wavelet transforms, health care, medical signal processing, motion artefact removals, wearable ECG, stationary wavelet transform, wearable electrocardiogram, SWT

## Abstract

Wearable Electrocardiogram (ECG) is attracting much attention in daily healthcare applications. From the viewpoint of long-term use, it is desired that the electrodes are non-contact with the human body. In this study, the authors propose an algorithm using the stationary wavelet transform (SWT) to remove motion artefact superimposed on ECG signal when using non-contact capacitively coupling electrodes. The authors evaluate the effect on motion artefact removal of this algorithm by applying it to various ECG signals with motion artefacts superimposed. As a result, the correlation coefficients of ECG signals with respect to the clean ones have been improved from 0.71 to 0.88 on median before and after motion artefact removal, which demonstrates the validity of the proposed SWT-based algorithm.

## 1 Introduction

In recent years, the demand on information and communication technology is increasing in healthcare and medical applications. Body area network (BAN) has been proposed for this purpose. BAN is a wireless network constructed by connecting various vital sensors on human body to collect and monitor health states in daily life [1, 2]. Wearable electrocardiogram (ECG) is one of typical vital sensors. By adding a wireless communication function in the wearable ECG, the ECG signal can be detected and sent to a coordinator of BAN in real time. Such a wearable ECG can be used to grasp the health state and to trigger an alarm at impending state of life. As an example, it may be used to monitor driver's ECG in an automobile.

Current ECG sensors usually employ gel electrodes that contact the skin directly. Although the gel electrodes are strong to noise, they are not suitable to long term use because of the deterioration of detection sensitivity with the drying of gel, allergic reaction to the person who has weak skin, and discomfort of contact. It is obvious that non-contact capacitively coupling electrodes are more promising for long term daily use. However, the body's movement may be easily superimposed on the detected ECG signal.

**Fig. 1:**
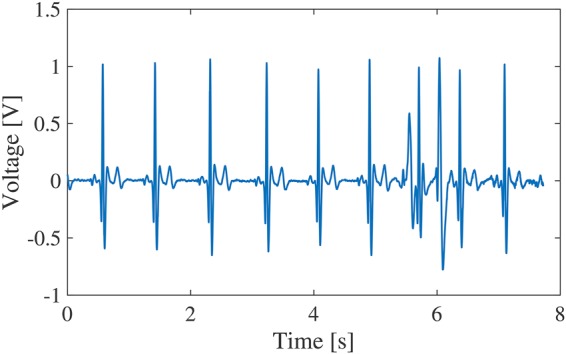
Measured ECG signal with motion artefact

Fig. 1 shows an exmaple of ECG signal with movement of upper body for a sitting person, measured by our developed human body communication-based wearable ECG [3, 4]. This wearable ECG employs human body as the communication medium and the detected ECG signal can be send to a receiver when the human hand touches it. It can be seen from Fig. 1 that the motion artefact due to the body's movement is superimposed in the ECG signal around 6 s. Its magnitude achieves a similar level to the QRS complex. Therefore, in order to realise a steady monitoring of ECG signal in daily life, it is necessary to effectively remove the motion artefact from the ECG signal.

An existing method to remove the motion artefact is to employ an accelerometer for measuring the body movement at the same time of ECG detection [5]. However, for non-contact electrode structure of ECG detection, an accelerometer directly attached to the human body is unacceptable. Another attempt is to employ stationary wavelet transform (SWT) to remove the motion artefact [6], where the QRS complex is extracted based on the energy of ECG signal. However, this method is difficult to work especially when the motion artefact occurs between two QRS complexes with a level similar to the R-wave. In this study, we propose an improved SWT-based algorithm to remove the motion artefact in ECG signal. We verify its validity in motion artefact removal by applying it to various ECG signals with superimposed body movement effect.

## 2 Algorithm

The wavelet transform of a signal *x*(*t*) is defined by

(1) $$W\left({b\comma \; a} \right)= \int_{ - \infty }^\infty x\left(t \right)\displaystyle{1 \over {\sqrt a }}\psi ^ \ast \left({\displaystyle{{t - b} \over a}} \right)\; {\rm d}t\eqno\lpar 1\rpar $$

Here, *a* is the scale parameter, *b* is the shift parameter, $\psi ^\ast \left(t \right)$ is the complex conjugate of mother wavelet $\psi \left(t \right)$ which satisfies the admissibility condition,

(2) $$C_\psi = 2\pi \int_{ - \infty }^\infty \displaystyle{{\vert \hat \psi \left(\omega \right)\vert ^2} \over \omega }\; {\rm d}\omega \eqno\lpar 2\rpar $$

where $\hat \psi \left(\omega \right)$ is Fourier transform of $\psi \left(t \right)$.

In the case of discrete wavelet transform, the scale parameter and the shift parameter take discrete value, $a = 2^j$, and $b = 2^jk$. The discrete wavelet transform of *x*(*t*) is defined by:

(3) $$W\left({k\comma \; j} \right)= \int_{ - \infty }^\infty x\left(t \right)\displaystyle{1 \over {\sqrt {2^j} }}\psi ^ \ast \left({2^{ - j}t - k} \right)\; {\rm d}t\eqno\lpar 3\rpar $$

**Fig. 2:**
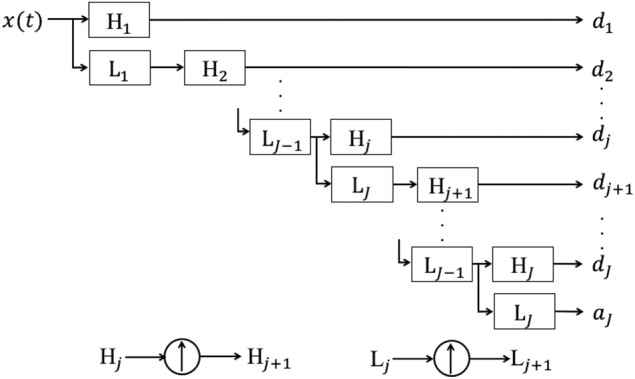
Stationary wavelet transform

**Fig. 3:**
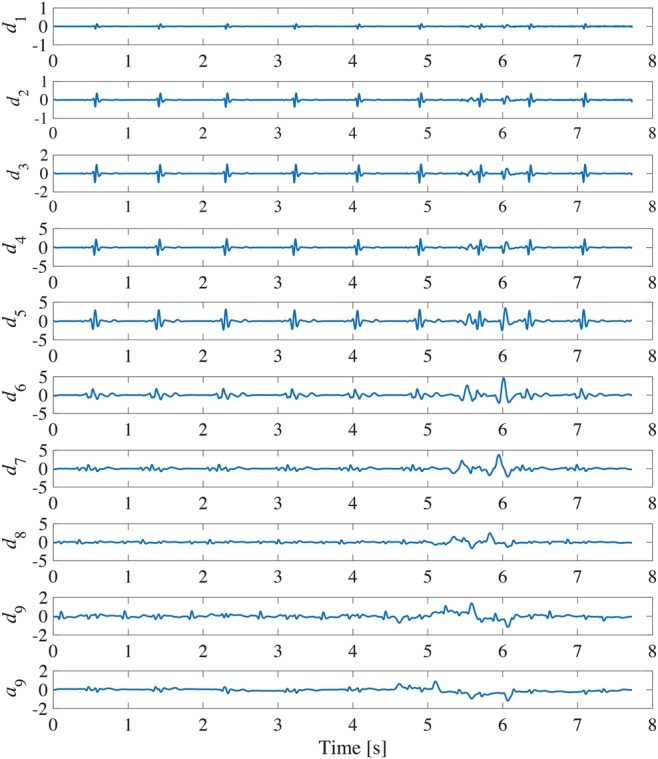
SWT of the ECG signal in Fig. 1

The discrete wavelet transform with Mallat devised algorithm can be obtained by combining high-pass filters $H_j$ and low-pass filters $L_j$ [7]. However, Mallat's algorithm carries out down-sampling. In the SWT [8], as shown in Fig. 2, by up-sampling the filter coefficients of the high-pass filters and the low-pass filters, it is possible to perform time-invariant wavelet transform. This is important in order to find outliers such as the artefact due to body movement and the specified signal components such as the QRS complex. The wavelet coefficients are given by the sequences $\lcub d_1\comma \; d_2\comma \; ...\comma \; d_J\rcub $ and the scaling coefficient is given by the sequence $a_J$. Where *J* represents the order of SWT. Since the ECG signal has a frequency component of 0–100 Hz, we set *J* = 9. Fig. 3 shows the SWT result of the ECG signal in Fig. 1. The main features of QRS complex are observed in the wavelet coefficients from $\lcub d_1\rcub $ to $\lcub d_6\rcub $.

**Fig. 4:**
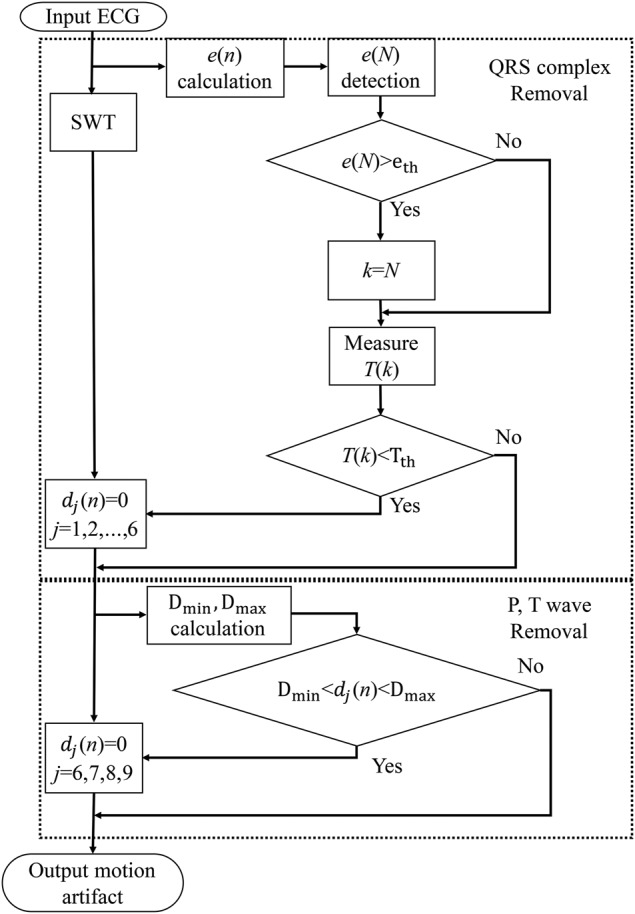
Flowchart of motion artefact extraction

**Fig. 5:**
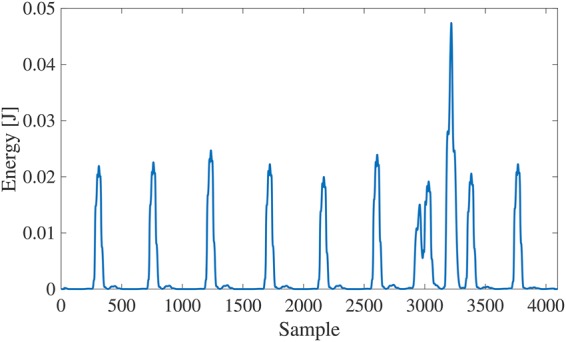
Calculated energy e(N) within per 0.1 s for the ECG signal in Fig. 1

Compared with the existing SWT-based algorithm only using the energy of ECG signal, we also consider the time periodicity of ECG signal and introduce it to the QRS complex detection part of the existing algorithm. The flowchart of the proposed algorithm for motion artefact removal is shown in Fig. 4. First of all, to detect the QRS complex from the energy of ECG signal, we calculate the energy *e*(*n*) of the ECG signal within a time period (0.1 s) and detect the local maximum value *e*(*N*) of the energy. Fig. 5 shows the calculated result of energy for the ECG signal in Fig. 1. The threshold $e_{{\rm th}}$ is determined from the value obtained by multiplying the median value of all $e\lpar N\rpar $ by α to avoid overlooking the QRS complex. If $e\lpar N\rpar \gt e_{{\rm th}}$, we define *k* = *N*, and calculate the time interval *T*(*k*) between *e*(*k*) and $e\lpar k - 1\rpar $. The threshold $T_{{\rm th}}$ is defined from the median value of *T*(*k*). Since the QRS complex should periodically appear in the ECG signal normally, if *T*(*k*) and *T*(*k* + 1) are both larger than $T_{{\rm th}}$, *e*(*k*) is determined as the energy of the QRS complex. On the other hand, the SWT is performed for the ECG signal. The Haar wavelet is used as the mother wavelet. Since the time width of the QRS complex is about 0.1 s, the wavelet coefficients $\lcub d_1\comma \; d_2\comma \; ...\comma \; d_6\rcub $ during the determined QRS complex period are replaced with 0. Next, P wave and T wave rise more slowly compared with the QRS complex. Hence, we focus on the wavelet coefficients $\lcub d_6\comma \; d_7\comma \; d_8\comma \; d_9\rcub $ that correspond to low-frequency component. Since the heart rate of an adult is 60–90 times within one minute usually, we detect the local maximum values and minimum values of wavelet coefficients $\lcub d_6\comma \; d_7\comma \; d_8\comma \; d_9\rcub $ every one second. The thresholds $D_{{\rm max}}$ and $D_{{\rm min}}$ are defined by the medians of the local maximum values and the local minimum values, respectively. Then, if $D_{{\rm min}} \lt d_j\lpar n\rpar \lt D_{{\rm max}}$, the wavelet coefficients $\lcub d_6\comma \; d_7\comma \; d_8\comma \; d_9\rcub $ during this time period are replaced with 0. Thereafter, by the inverse SWT, the motion artefact can be extracted, and then we can remove the motion artefact from the ECG signal by subtracting the extracted one.

## 3 Results

**Fig. 6:**
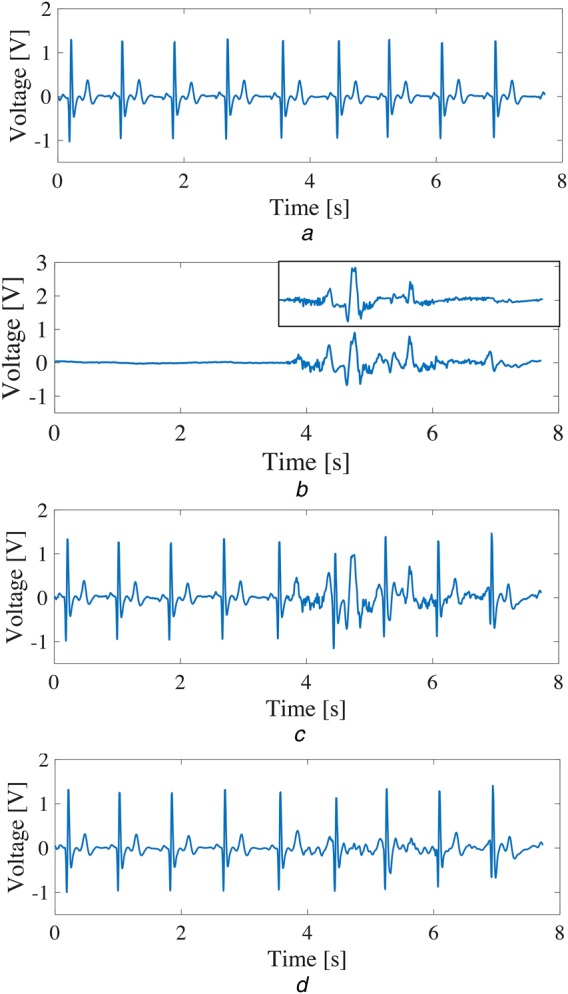
Artificial ECG signal by superimposing a motion artefact and measured clean ECG signal *a* Clean ECG signal *b* Motion artefact *c* ECG signal with superimposed motion artefact *d* Motion artefact removal from the signal in Fig. 6*c* using the proposed algorithm

To verify the validity of the proposed algorithm, we produced an artificial ECG signal by superimposing a motion artefact as shown in Fig. 6*b* to a measured clean ECG signal in Fig. 6*a*. We assumed that the motion artefact was caused by electrode slippage and obtained the motion artefact by attaching the electrode on the arm and moving it up and down. Fig. 6*c* shows the artificial ECG signal with superimposed motion artefact. Comparing the two waveforms in Figs. 6*c* and 1, it can be said that the artificial ECG waveform with superimposed motion artefact is similar to the actual one. Fig. 6*d* shows the result after applying the algorithm to remove the motion artefact with the factor *α* = 0.8 for the threshold $e_{{\rm th}}$. As can be seen from Fig. 6*d*, the motion artefact has been significantly removed from the ESG signal without obvious degradation of signal quality. Moreover, the extracted motion artefact waveform by the proposed algorithm is shown on the upper right of Fig. 6*b*. It can be confirmed that the extracted motion artefact is almost the same as the original one.

**Fig. 7:**
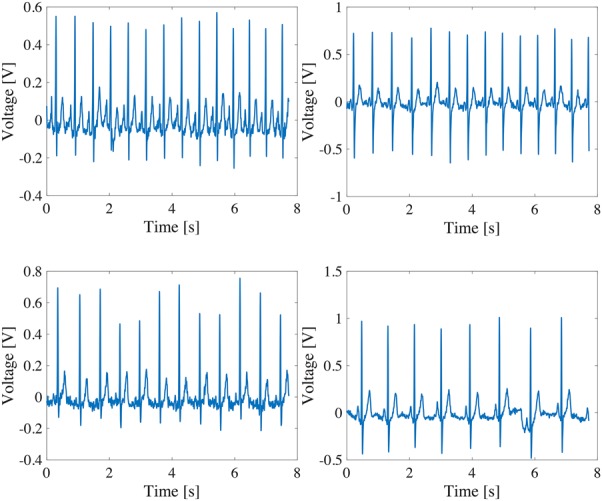
Four kinds of clean ECG signals selected from the ECG-ID database

**Fig. 8:**
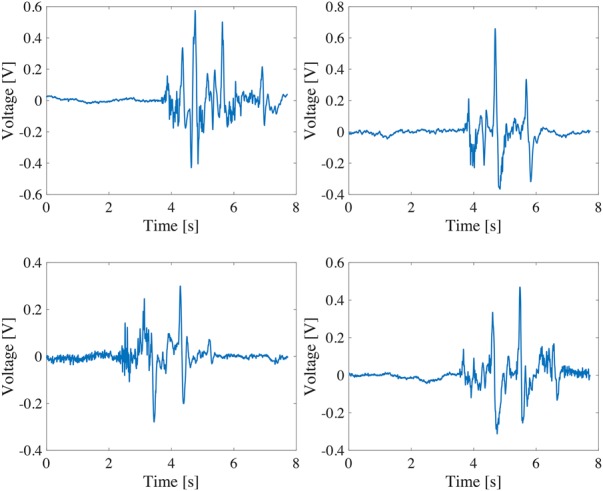
Four kinds of measured motion artefact

**Fig. 9:**
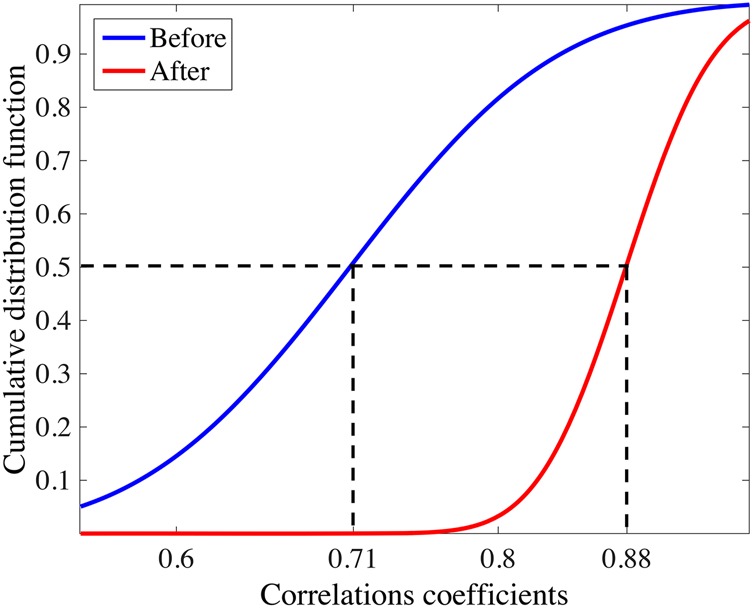
Cumulative distribution of the correlation coefficients before and after applying our algorithm for various ECG signals

A quantitative evaluation was conducted by calculating the correlation coefficients between the ECG signal after motion artefact removal and the clean ECG signal. Compared with the correlation coefficient of 0.76 between Figs. 6*a* and *c*, the correlation coefficient has been improved to 0.93 between Figs. 6*a* and *d*. In addition, we produced 16 artificial ECG signals by superimposing four measured motion artefact as shown in Fig. 8 on four clean ECG signals as shown in Fig. 7. The four clean ECG signals were selected from the ECG-ID database [9, 10] (Fig. 8). Our algorithm was quantitatively evaluated by the correlation coefficients between the ECG signals after motion artefact removal and the clean ECG signals. Fig. 9 shows the cumulative distribution of the correlation coefficients before and after applying the algorithm for the 16 ECG signals with motion artefacts. In all of the cases, the motion artefacts have been significantly removed so that the correlation coefficient has been improved from 0.71 to 0.88 on median.

Next, we investigated the optimal value of the factor α for the threshold $e_{{\rm th}}$ which detects the QRS complex. We applied our algorithm to 20 different ECG signals selected from the ECG-ID database. By comparing the number of *R* waves, we determined the appropriate *α* for each ECG signal and made their average as the optimal value of *α*. As a result, *α* = 0.6 is likely most suitable as the optimal value.

**Fig. 10:**
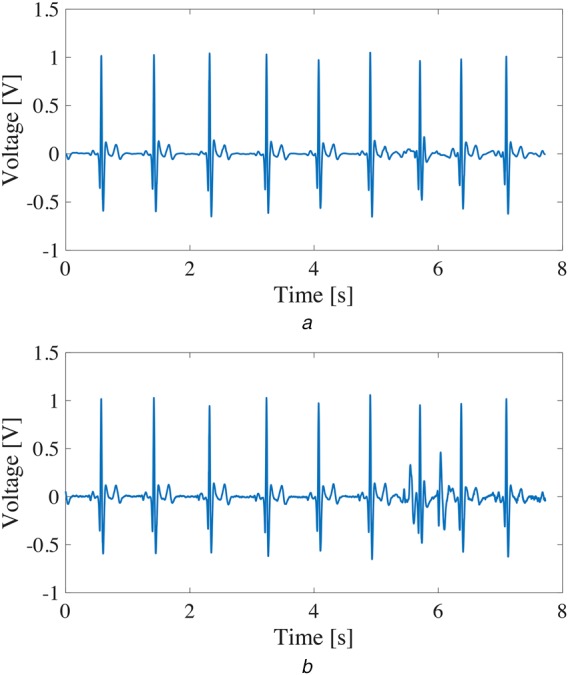
ECG waveform after applying our algorithm and the ECG waveform after applying their algorithm *a* Motion artefact removal using our algorithm *b* Motion artefact removal using Strasser *et al.*'s algorithm

To illustrate the performance of our algorithm, we also compared the motion artefact removal effect by applying our algorithm and Strasser *et al.*'s algorithm [6] to the ECG signal in Fig. 1. The ECG waveform after applying our algorithm is shown in Fig. 10*a*, and the ECG waveform after applying their algorithm is shown in Fig. 10*b*. It can be confirmed that the motion artefact has been more significantly removed by our algorithm. In addition, we examine whether it is possible to remove motion artefact in ECG signals including premature ventricular contraction (PVC). We superimposed the motion artefacts as shown in Fig. 8 on several ECG signals with the PVC, selected from the MIT-BIH Arrhythmia Database [10, 11]. We attempted to apply our algorithm to these ECG signals, but it did not work well in removing the motion artefacts. This may be due to that our algorithm requires the time interval of QRS complex.

## 4 Conclusion

Wearable ECG integrated with non-contact ECG detection and human body communication can provide a lot of convenience in daily monitoring of ECG signal. In this study, we paid attention to the motion artefact in the ECG signal measured by our wearable ECG, and proposed a SWT-based algorithm for motion artefact removal. To evaluate the validity of the motion artefact removal algorithm, we applied it to various ECG signals with motion artefact superimposed artificially. Then we calculated the correlation coefficients between the ECG signals before and after the motion artefact removal and the clean ECG signals, respectively. It is found that the correlation coefficients have been improved from 0.71 to 0.88 on median before and after the motion artefact removal, which demonstrates the validity of the proposed SWT-based algorithm.

The future work is to investigate the influence of other mother wavelet on motion artefact removal and to devise an algorithm which is valid even in the existence of a PVC beat.

## 5 Funding and declaration of interests

This work was supported in part by JSPS Grants-in-Aid for Scientific Research Grant Number 15H04006. Conflict of interest: None declared.
